# The genus *Aerophilus* Szépligeti, 1902 (Hymenoptera, Braconidae, Agathidinae) in China

**DOI:** 10.3897/BDJ.13.e157012

**Published:** 2025-06-13

**Authors:** Qiong Wu, Pu Tang, Yu Fang, Cornelis van Achterberg, Xue-xin Chen

**Affiliations:** 1 State Key Laboratory of Rice Biology, Institute of Insect Sciences, Zhejiang University, Hangzhou, China State Key Laboratory of Rice Biology, Institute of Insect Sciences, Zhejiang University Hangzhou China; 2 Zhejiang Provincial Key Lab of Biology of Crop Pathogens and Insects, Institute of Insect Sciences, Zhejiang University, Hangzhou, China Zhejiang Provincial Key Lab of Biology of Crop Pathogens and Insects, Institute of Insect Sciences, Zhejiang University Hangzhou China; 3 National Demonstration Center for Experimental Agrobiology Education, Zhejiang University, Hangzhou, China National Demonstration Center for Experimental Agrobiology Education, Zhejiang University Hangzhou China; 4 Institute of Insect Sciences, Zhejiang University, Hangzhou, China Institute of Insect Sciences, Zhejiang University Hangzhou China

**Keywords:** *Lytopylus*, new record, new synonym, identification key

## Abstract

**Background:**

The genus *Aerophilus* Szépligeti, 1902 (Hymenoptera, Braconidae, Agathidinae) is distributed throughout the globe, attacking caterpillars from multiple families within the Lepidoptera. Two species of *Aerophilus* were recorded from China prior to this study.

**New information:**

Four Chinese species of *Aerophilus* Szépligeti, 1902 are recognised. Two new species, *A.brevicaudis* sp. nov. and *A.convexus* sp. nov., are described and illustrated. A species, *A.rufipes* (Nees, 1812), is recorded from China for the first time. A new synonym is proposed, *A.ebulus* (Nixon, 1950) with *A.romani* (Shestakov, 1940). A key to Chinese species of the genus *Aerophilus* is provided.

## Introduction

The genus *Aerophilus* Szépligeti, 1902 (Hymenoptera, Braconidae, Agathidinae) is a distinctive and ecologically fascinating member of the Agathidinae. The diversity of its hosts is remarkable, as seen in the overview of Costa Rican species by Sharkey et al. (2011), ten species having been reared from five host families — Crambidae, Elachistidae, Pyralidae, Thyrididae and Tortricidae.

Historically, the genus *Lytopylus* Foerster, 1863, was considered a junior synonym of *Bassus* Fabricius, *Microdus* Nees and *Agathis* Latreille for a long time (Yu et al. 2016). Recently, Sharkey et al. (2009) reinstated it from synonymy in their revision of the Oriental genera of Agathidinae by the sculpture of the third metasomal tergite and by the structure of the propodeal foramen. An important additional character is the shallow transverse groove of the third tergite. Sharkey et al. (2009) synonymised the genus *Facilagathis* van Achterberg & Chen, 2004 with *Lytopylus*, subsequently including the two Chinese species, *F.spinulata* van Achterberg & Chen, 2004 and *F.macrocentroides* van Achterberg & Chen, 2004 in *Lytopylus*. This synonymy of *Facilagathis* with *Lytopylus* and, subsequently, with *Aerophilus* is not accepted because of the presence of the numerous spiny pegs on the hind basitarsus ventrally (absent in *Aerophilus*), the very slender first metasomal tergite and hind coxa (shape of tergite and coxa normal in *Aerophilus*) and the absence of a distinct trace of vein 2-CU of the hind wing (present in *Aerophilus*).

After this new concept was proposed, several papers on the genus *Lytopylus* were published (van Achterberg and Long 2010, van Achterberg 2011, Sharkey and Clutts 2011, Sharkey et al. 2011, Stevens et al. 2011). Later, Sharkey et al. (2016) transferred all species previously included under *Lytopylus* to *Aerophilus* and established *Aerophilus* as the correct name for the group.

Two species of *Aerophilus* were recorded from China prior to this study, *A.ebulus* (Nixon) and *A.romani* (Shestakov) (Chou and Sharkey 1989, Chen and Yang 2006, Yu et al. 2016).

During our study of Chinese braconids, we discovered four species, *A.brevicaudis* Tang & Chen, sp. nov., *A.convexus* Tang & Chen, sp. nov., *A.romani* and *A.rufipes* (Nees). *Aerophilusebulus* is proposed as a new junior synonym of *A.romani*. In the present paper, the new species are described and illustrated and a key to Chinese species of *Aerophilus* is provided.

## Materials and methods

This study is based on the specimens preserved in the Parasitic Hymenoptera Collection of Institute of Insect Sciences, Zhejiang University (ZJUH).

The terminology and measurements used follow van Achterberg (1993). All descriptions and measurements were made under a Zeiss Stemi 2000-C microscope; a digital microscope (Keyence VHX–7000) was used for the photos. Type specimens and other materials are deposited in the Parasitic Hymenoptera Collection of the Zhejiang University, Hangzhou, China (ZJUH).

## Taxon treatments

### Aerophilus brevicaudis

Wu & Tang sp. nov.

BC7CF2BF-146C-5A86-9E33-64C984A80849

D53D67DE-57CA-4E05-A49E-1CE3301404B8

#### Materials

- **Type status:** Holotype. **Occurrence:** catalogNumber: 200614937 (ZJUH); recordedBy: Zhang Hongying; individualCount: 1; sex: female; lifeStage: adult; occurrenceID: 4F4BDE06-9929-5E6C-B154-AEA421476243; **Location:** country: China; stateProvince: Sichuan; county: Pingwu; locality: Baimazhai; **Event:** verbatimEventDate: 25.VII.2006; **Record Level:** basisOfRecord: PreservedSpecimen
- **Type status:** Paratype. **Occurrence:** catalogNumber: 200612454, 200614933, 200615100, 200615084, 200612448, 200612498, 200615056, 200615067, 200615141, 200614805, 200614313, 200614765, 200614931, 200615000, 200615057, 200615194, 20061486 (ZJUH); recordedBy: Zhang Hongying; individualCount: 17; sex: female; lifeStage: adult; occurrenceID: CA219E10-4B65-5975-A1DB-EAB4400898B0; **Location:** country: China; stateProvince: Sichuan; county: Pingwu; locality: Baimazhai; **Event:** verbatimEventDate: 25.VII.2006; **Record Level:** basisOfRecord: PreservedSpecimen
- **Type status:** Paratype. **Occurrence:** catalogNumber: 200612482, 200615039, 200612599, 200614988, 200614800, 200614848 (ZJUH); recordedBy: Zhang Hongying; individualCount: 6; sex: female; lifeStage: adult; occurrenceID: 63D0F218-F51B-5B2B-AE7E-B496330E3EB8; **Location:** country: China; stateProvince: Sichuan; county: Pingwu; locality: Baimazhai; **Event:** verbatimEventDate: 25.VII.2006; **Record Level:** basisOfRecord: PreservedSpecimen
- **Type status:** Paratype. **Occurrence:** catalogNumber: 200614143, 200614138, 200612570, 200614373, 200612686, 200614206 (ZJUH); recordedBy: Gao Zhilei; individualCount: 6; sex: female; lifeStage: adult; occurrenceID: 1AC64E95-5BB6-5A2C-A23E-B15B7F748648; **Location:** country: China; stateProvince: Sichuan; county: Pingwu; locality: Baimazhai; **Event:** verbatimEventDate: 25.VII.2006; **Record Level:** basisOfRecord: PreservedSpecimen
- **Type status:** Paratype. **Occurrence:** catalogNumber: 200612645, 200612634, 200614278, 200614245, 200612601, 200612537, 200613516 (ZJUH); recordedBy: Gao Zhilei; individualCount: 7; sex: female; lifeStage: adult; occurrenceID: 3F8447BA-A9FB-5126-873A-148F7E6DEB8A; **Location:** country: China; stateProvince: Sichuan; county: Pingwu; locality: Baimazhai; **Event:** verbatimEventDate: 25.VII.2006; **Record Level:** basisOfRecord: PreservedSpecimen

#### Description

Holotype, female, length of body 6.7 mm, of fore wing 4.0 mm.

**Figure 1.**
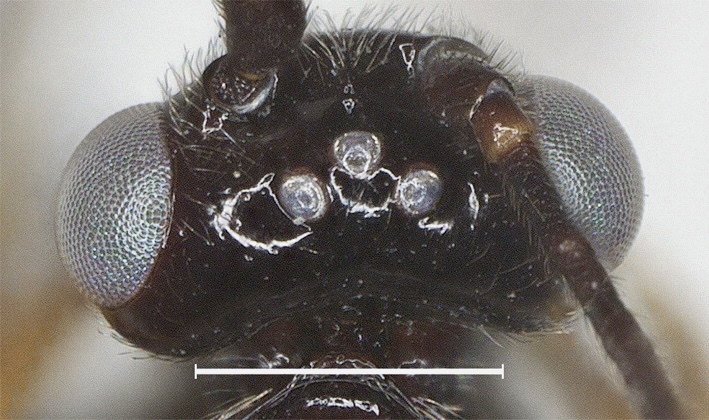
*Aerophilusbrevicaudis* Wu & Tang, sp. nov., holotype. Head, dorsal aspect. Scale-bar 1 mm.

**Figure 2.**
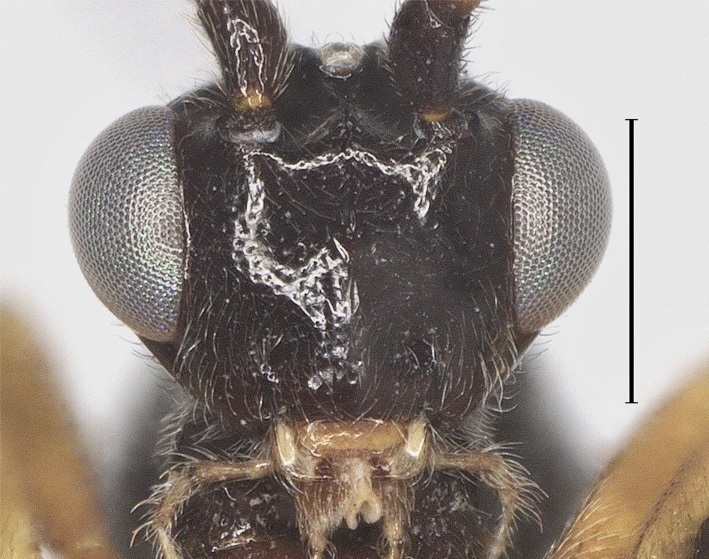
*Aerophilusbrevicaudis* Wu & Tang, sp. nov., holotype. Head, front aspect. Scale-bar 1 mm.

**Figure 3.**
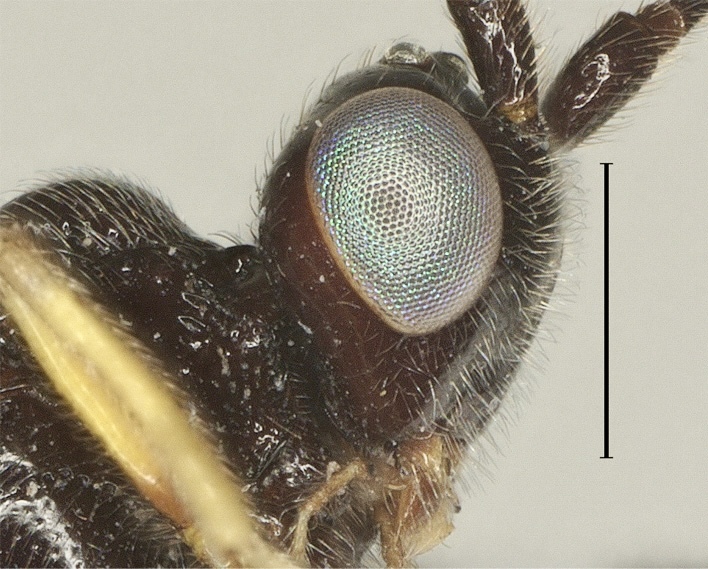
*Aerophilusbrevicaudis* Wu & Tang, sp. nov., holotype. Head, lateral aspect. Scale-bar 1 mm.

**Head.** Antennal segments 33, length of third flagellomere 1.2 times fourth flagellomere, length of third, fourth and penultimate flagellomere 4.0, 3.3 and 2.3 times their width, respectively; length of apical antennal flagellomere 1.4 times as long as penultimate flagellomere; maxillary palp 0.6 times height of head; malar space 2.5 times as long as basal width of mandible; in dorsal view, length of eye 2.5 times temple; ocelli in low triangle, POL:OD:OOL= 7:6:11 (Fig. 1); face shiny and distinctly rather finely punctate (Fig. 2); frons with weak medial ridge, shiny with sparse fine punctures; vertex and temple shiny with sparse fine punctures (Fig. 3).

**Figure 4.**
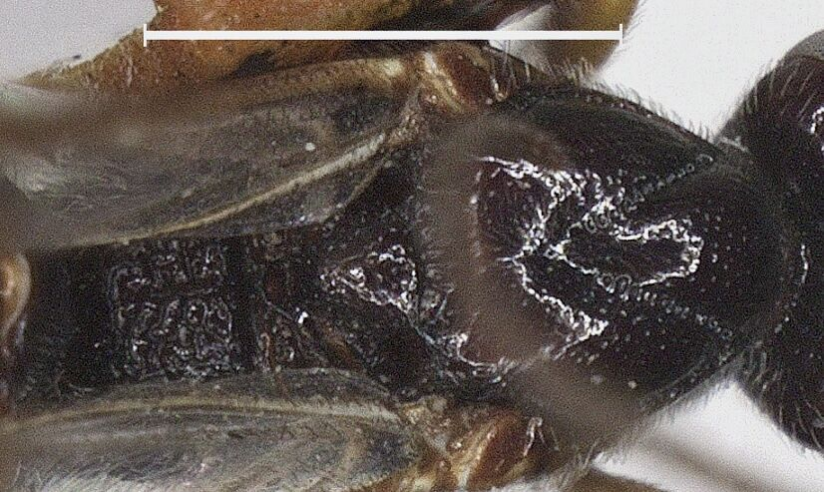
*Aerophilusbrevicaudis* Wu & Tang, sp. nov., holotype. Mesosoma, dorsal aspect. Scale-bar 1 mm.

**Figure 5.**
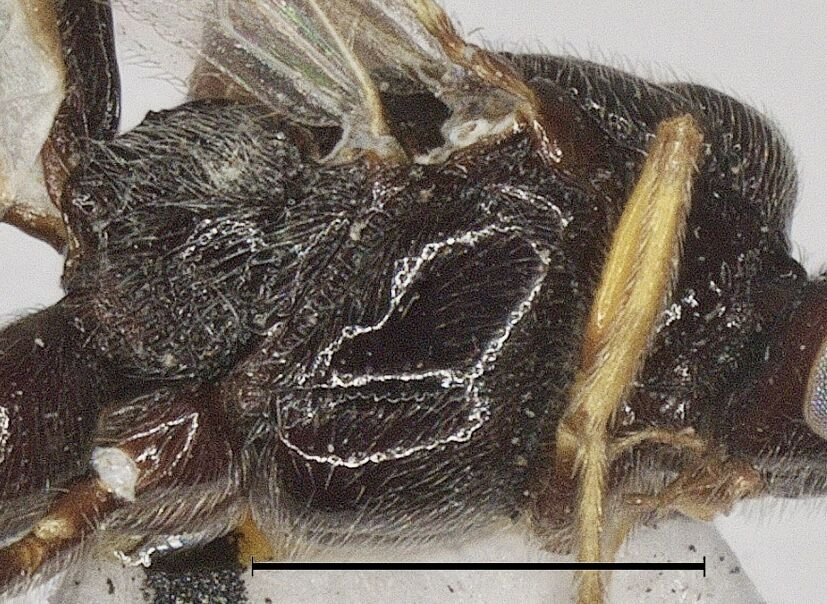
*Aerophilusbrevicaudis* Wu & Tang, sp. nov., holotype. Mesosoma, lateral aspect. Scale-bar 1 mm.

**Mesosoma.** Length of mesosoma 1.5 times its height; pronotum smooth with carinae anteriorly, finely densely punctate dorso-posteriorly and posterior groove almost smooth; area near lateral carina of mesoscutum crenulate; mesoscutum shiny, sparsely punctate and setose; notauli complete and narrowly crenulate; scutellar sulcus 0.5 times as long as scutellum with 3 carinae; scutellum without subposterior crest, sparsely, but distinctly punctate (Fig. 4); precoxal sulcus weakly crenulate and narrow; mesopleuron below precoxal sulcus with sparse fine punctures; mesopleuron above precoxal sulcus smooth; metapleuron densely setose, spaced moderately punctate and ventrally rugose (Fig. 5); propodeum reticulate-rugose with a median longitudinal carina in basal half.

**Figure 6.**
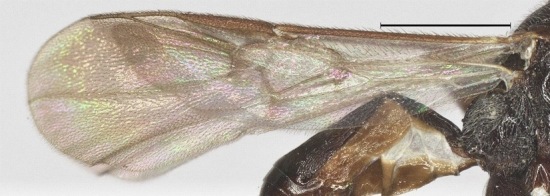
*Aerophilusbrevicaudis* Wu & Tang, sp. nov., holotype. Fore wing. Scale-bar 1 mm.

**Wings.** Fore wing: second submarginal medium-sized and triangular; marginal cell narrow; vein SR1 straight; r:3-SR+SR1 = 3:35. Hind wing: vein M+CU 0.7 times as long as vein 1-M (13:19) (Fig. 6).

**Figure 7.**
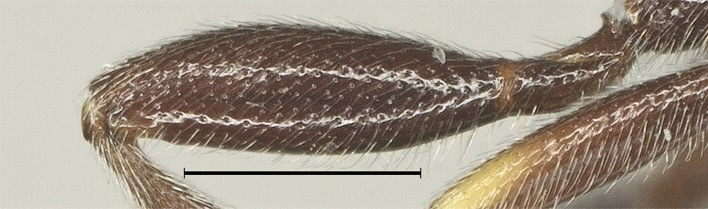
*Aerophilusbrevicaudis* Wu & Tang, sp. nov., holotype. Hind femur. Scale-bar 1 mm.

**Legs.** Length of hind femur, tibia and basitarsus 3.2, 5.2 and 6.5 times their width, respectively; hind femur (as remainder of legs) with short setae (Fig. 7l); length of outer and inner spur of middle tibia 0.4 and 0.6 times middle basitarsus, respectively; outer side of middle tibia with 8 pegs; length of outer and inner spur of hind tibia 0.3 and 0.5 times hind basitarsus, respectively; tarsal claws with lobe.

**Figure 8.**
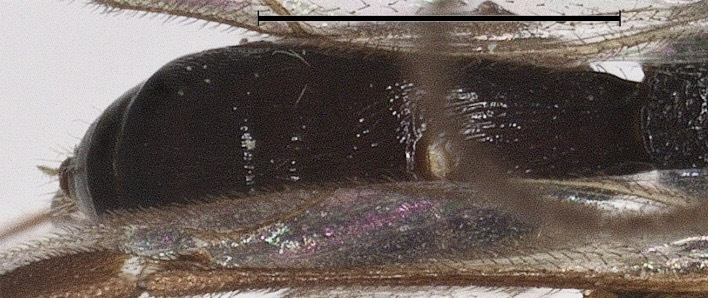
*Aerophilusbrevicaudis* Wu & Tang, sp. nov., holotype. Metasoma, dorsal aspect. Scale-bar 1 mm.

**Figure 9.**
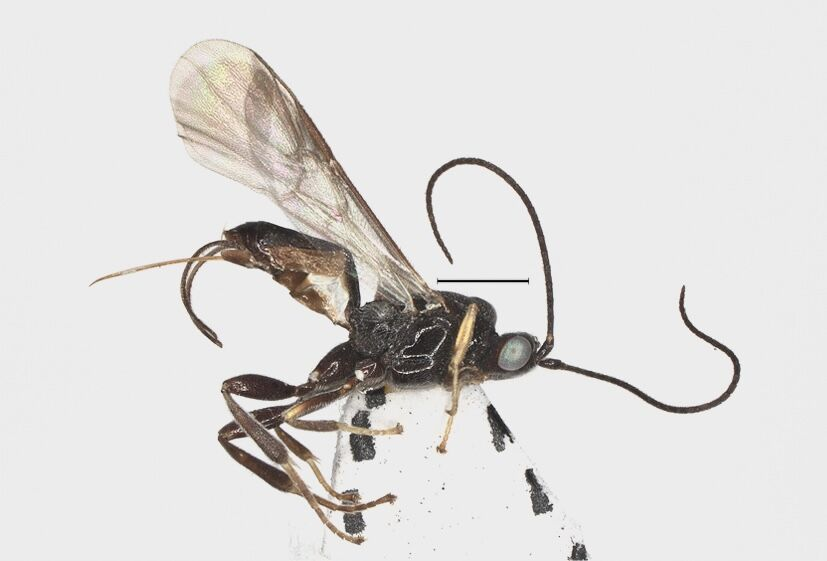
*Aerophilusbrevicaudis* Wu & Tang, sp. nov., holotype. Habitus, lateral aspect. Scale-bar 1 mm.

**Metasoma.** Length of first tergite 1.2 times its apical width; first tergite coarsely longitudinally striate; second tergite 1.1 times as long as third tergite, coarsely longitudinally striate with transverse groove; third tergite coarsely longitudinally striate in basal 0.7, smooth in apical 0.3; remainder of metasoma smooth (Fig. 8); Setose portion of the ovipositor sheath 0.5 times as long as fore wing (Fig. 9).

**Colour.** Black; fore leg (but coxa, trochanter, femur partly dark brown), middle tarsus and base of hind tarsus brownish-yellow; pterostigma dark brown; wing membrane hyaline, very faintly infuscate in apical fifth (Fig. 9).

**Variation.** Antennal segments 30–34; outer side of middle tibia with row of 5–7 pegs; length of hind femur 3.0–3.2 times as long as wide; length of body 6.2–7.1 mm, of fore wing 3.9–4.3 mm; fore and middle legs, hind tarsus from brownish-yellow to dark brown in most part.

#### Diagnosis

From “brev” (Latin for “short”) and “caud” (Latin for “tail”), because of the short ovipositor sheath.

#### Etymology

This new species is very similar to *L.romani* (Shestakov, 1940), but differs in having the ovipositor sheath distinctly shorter, 0.5 times as long as fore wing; wing membrane almost hyaline; and vein 1-R1 of fore wing distinctly longer than 2-R1.

#### Distribution

China (Sichuan)

#### Biology

Unknown.

### Aerophilus convexus

Wu & Tang sp. nov.

A4DC5E72-5BA9-5BF3-A935-27A954079435

5B0CC95A-07A7-4438-AE1A-246C84F3B133

#### Materials

- **Type status:** Holotype. **Occurrence:** catalogNumber: 801993 (ZJUH); recordedBy: He Junhua; individualCount: 1; sex: female; lifeStage: adult; occurrenceID: 3C37DC2C-2C61-56B5-A913-D2B877D82D01; **Location:** country: China; stateProvince: Sichuan; county: Guanxian; **Event:** verbatimEventDate: 4.VIII.1980; **Record Level:** basisOfRecord: PreservedSpecimen

#### Description

Holotype, ♀, length of body 4.6 mm, of fore wing 3.9 mm.

**Figure 10.**
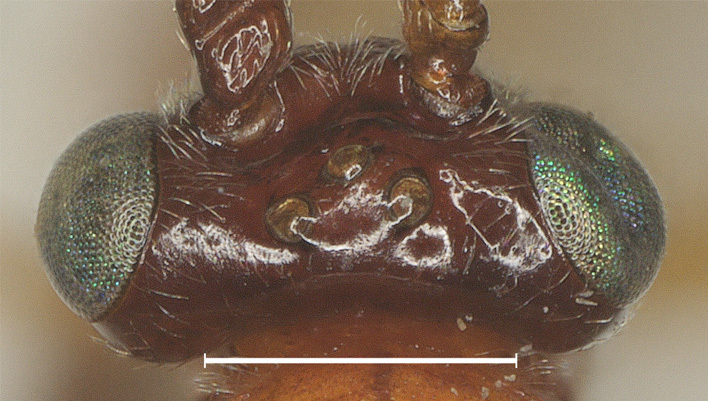
*Aerophilusconvexus* Wu & Tang, sp. nov., holotype. Head, dorsal aspect. Scale-bar 1 mm.

**Figure 11.**
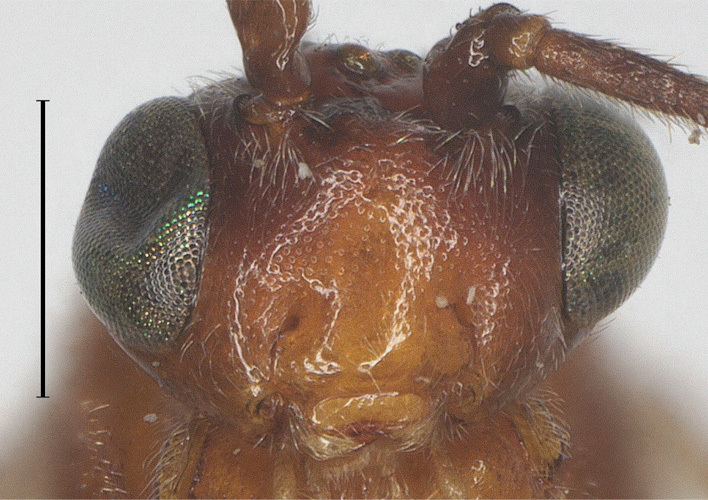
*Aerophilusconvexus* Wu & Tang, sp. nov., holotype. Head, front aspect. Scale-bar 1 mm.

**Figure 12.**
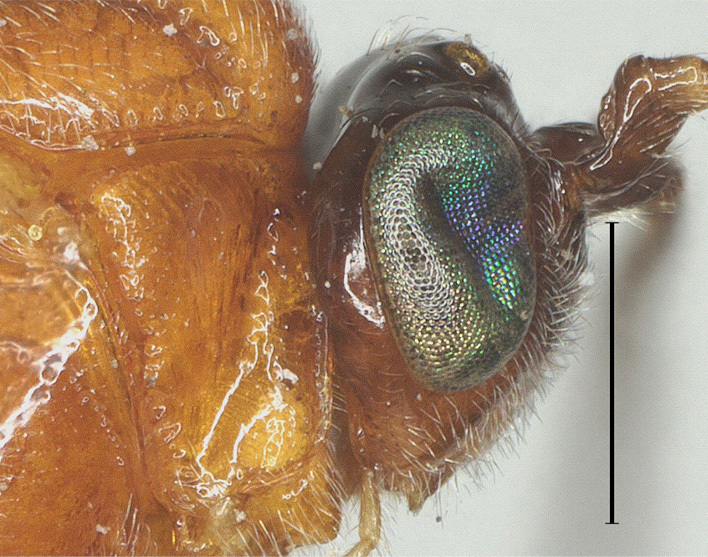
*Aerophilusconvexus* Wu & Tang, sp. nov., holotype. Head, lateral aspect. Scale-bar 1 mm.

**Head.** Antennal segments 35, length of third flagellomere 1.2 times fourth flagellomere, length of third and fourth flagellomere 3.0 and 2.5 times their width, respectively; maxillary palp 0.8 times height of head; malar space 1.5 times as long as basal width of mandible; in dorsal view length of eye 2.1 times temple (Fig. 10); face distinctly punctate (Fig. 11); frons without medial ridge, smooth; vertex and temple smooth (Fig. 12).

**Figure 13.**
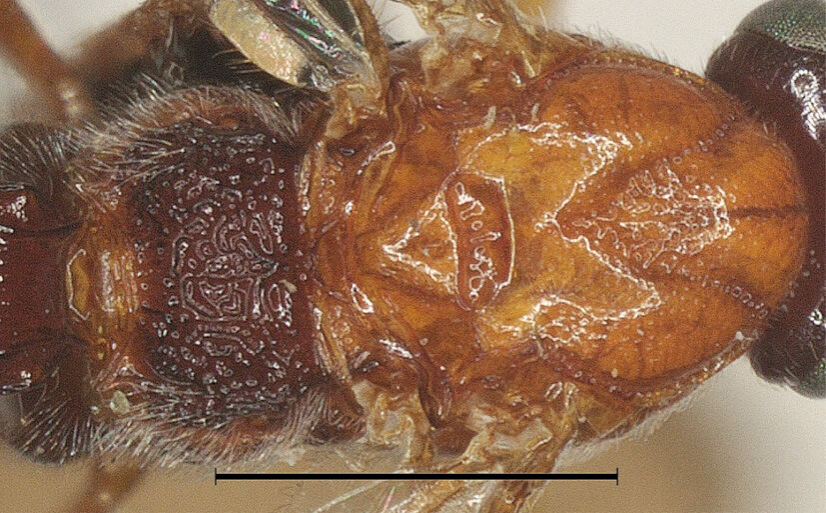
*Aerophilusconvexus* Wu & Tang, sp. nov., holotype. Mesosoma, dorsal aspect. Scale-bar 1 mm.

**Figure 14.**
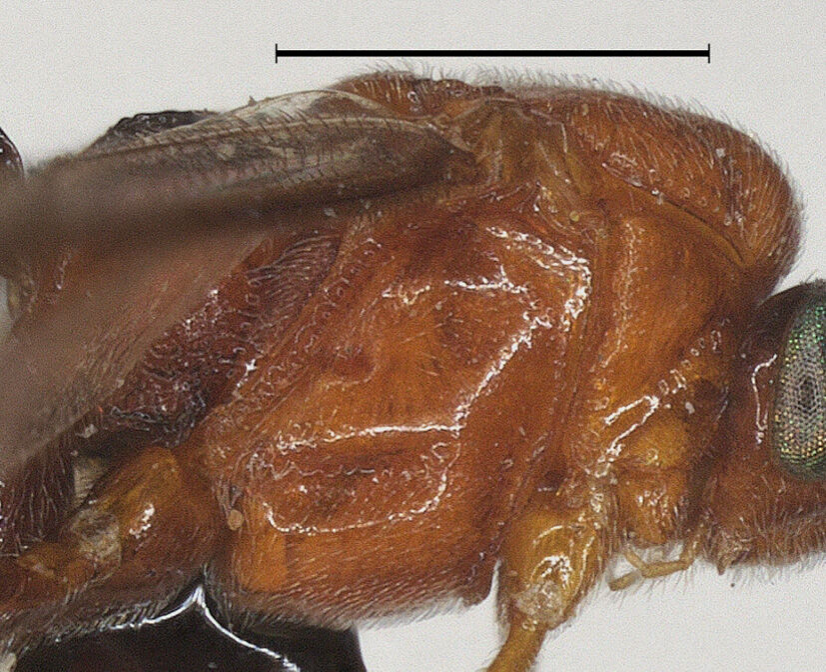
*Aerophilusconvexus* Wu & Tang, sp. nov., holotype. Mesosoma, lateral aspect. Scale-bar 1 mm.

**Mesosoma.** Length of mesosoma 1.2 times its height; pronotum finely punctate dorso-posteriorly and posterior groove finely crenulate; area near lateral carina of mesoscutum crenulate; mesoscutum densely punctate and setose; notauli complete and narrowly crenulate; scutellar sulcus 0.5 times as long as scutellum with 3 carinae; scutellum without subposterior crest, shiny, sparsely punctate (Fig. 13); precoxal sulcus weakly crenulate and narrow; mesopleuron below precoxal sulcus with sparse fine punctures; mesopleuron above precoxal sulcus mostly smooth, finely punctate anteriorly; metapleuron densely setose, spaced moderately punctate and ventrally rugose (Fig. 14); propodeum reticulate-rugose (Fig. 13).

**Figure 15.**
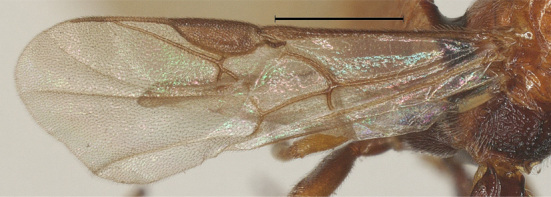
*Aerophilusconvexus* Wu & Tang, sp. nov., holotype. Fore wing. Scale-bar 1 mm.

**Wings.** Fore wing: second submarginal medium-sized and triangular; marginal cell narrow; vein SR1 straight; r:3-SR+SR1=3:46 (Fig. 15). Hind wing: vein M+CU 0.9 times as long as vein 1-M (14:16).

**Figure 16.**
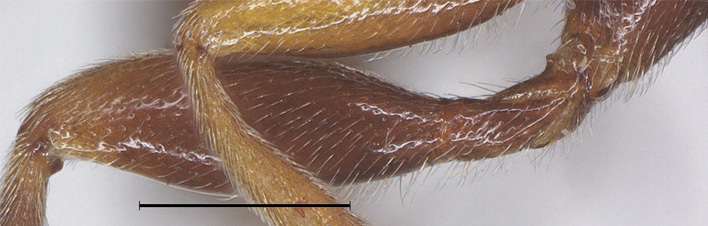
*Aerophilusconvexus* Wu & Tang, sp. nov., holotype. Hind femur. Scale-bar 0.5 mm.

**Legs.** Length of hind femur, tibia and basitarsus 3.1, 5.5 and 8.5 times their width, respectively; hind femur (as remainder of legs) with short setae (Fig. 16); length of outer and inner spur of middle tibia 0.4 and 0.6 times middle basitarsus, respectively; outer side of middle tibia with 12 pegs; length of outer and inner spur of hind tibia 0.3 and 0.5 times hind basitarsus, respectively; tarsal claws with lobe.

**Figure 17.**
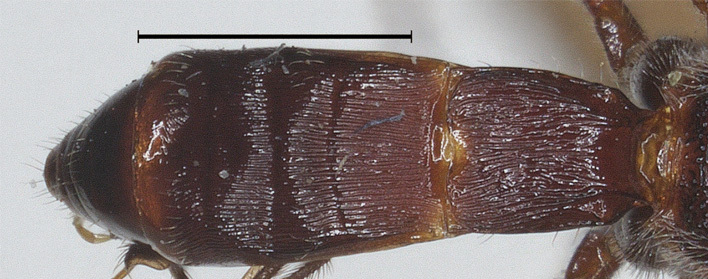
*Aerophilusconvexus* Wu & Tang, sp. nov., holotype. Metasoma, dorsal aspect. Scale-bar 1 mm.

**Metasoma.** Length of first tergite 1.2 times its apical width; first tergite coarsely longitudinally striate; second tergite 1.15 times as long as third tergite, coarsely longitudinally striate with transverse groove; third tergite coarsely longitudinally striate in basal 0.7, smooth in apical 0.3; remainder of metasoma smooth (Fig. 17); ovipositor sheath 0.6 times as long as fore wing.

**Figure 18.**
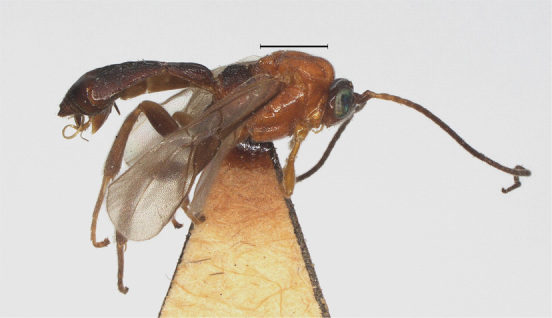
*Aerophilusconvexus* Wu & Tang, sp. nov., holotype. Habitus, lateral aspect. Scale-bar 1 mm.

**Colour.** Black; mandible, palpi, pronotum, mesoscutum, scutellum and mesopleuron orange-brown; head ventrally half, fore and middle legs brownish-yellow (but middle coxa, trochanter, trochantellus and femur partly dark brown); hind leg almost entirely dark brown; pterostigma dark brown; wing membrane subhyaline (Fig. 18).

#### Diagnosis

This new species is very similar to *L.romani* (Shestakov, 1940), but differs in having the mesoscutum distinctly protruding forward; frons without a medial ridge; and length of mesosoma 1.2 times its height.

#### Etymology

From “convexus” (Latin for “convex”), because of the convex mesoscutum.

#### Distribution

China (Sichuan)

#### Biology

Unknown.

### Aerophilus romani

(Shestakov 1940)

504AF055-582E-55E8-A66F-9AA743BC57C8

Microdus romani Shestakov 1940: 14.
Agathis romani : *Shenefelt 1970*: 351.
Bassus romani : *Sharkey 1998*: 528; Chen and Yang 2006: 87.
Lytopylus romani : van Achterberg and Long 2010: 93; Sharkey and Clutts 2011: 127.
Bassus ater Chou and Sharkey 1989: 155. Synonymised by Sharkey (1998).
Agathis burmensis Bhat and Gupta 1977: 142. Synonymised by Sharkey et al. (2011), Sharkey and Clutts (2011) and Sharkey and Clutts (2011).
*Agathisebula* Nixon 1950: 469.
Bassus ebulus : *Chou and Sharkey 1989*: 158. Synonymised by Sharkey (1998).
Lytopylus ebulus : *Sharkey and Clutts 2011*: 126 (reinstated).
Aerophilus ebulus : *Sharkey et al. 2016*: 54. **syn. n.**

#### Materials

- **Type status:** Holotype. **Occurrence:** lifeStage: adult; occurrenceID: A9A598D5-638B-543C-BD2D-3DA45D112B1A; **Taxon:** scientificName: Agathisebula Nixon, 1950; **Record Level:** basisOfRecord: PreservedSpecimen
- **Type status:** Other material. **Occurrence:** catalogNumber: 20004545; 20004518; 20004569; 20004533(ZJUH); recordedBy: Lin Naiquan; individualCount: 4; sex: female; lifeStage: adult; occurrenceID: CB150999-0177-5586-A9B5-1FD2B96D4621; **Location:** country: China; stateProvince: Liaoning; municipality: Shenyang; locality: Dongling; **Event:** verbatimEventDate: 9.VII.1992; **Record Level:** basisOfRecord: PreservedSpecimen
- **Type status:** Other material. **Occurrence:** catalogNumber: 947620; 947672(ZJUH); recordedBy: Lou Juxian; individualCount: 2; sex: female; lifeStage: adult; occurrenceID: 60D59A79-EEFE-5E49-9476-4674FA6D9A6D; **Location:** country: China; stateProvince: Liaoning; municipality: Shenyang; locality: Dongling; **Event:** verbatimEventDate: 21.VI.1994; **Record Level:** basisOfRecord: PreservedSpecimen
- **Type status:** Other material. **Occurrence:** catalogNumber: 821523; 822287 (ZJUH); recordedBy: He Junhua; individualCount: 2; sex: female; lifeStage: adult; occurrenceID: B77CEF16-337B-5B0F-B480-A2C93C49BD65; **Location:** country: China; stateProvince: Guangxi; county: Longzhou; locality: Nonggang; **Event:** verbatimEventDate: 19.V.1982; **Record Level:** basisOfRecord: PreservedSpecimen
- **Type status:** Other material. **Occurrence:** catalogNumber: 882682 (ZJUH); recordedBy: Guo Shijian; individualCount: 1; sex: female; lifeStage: adult; occurrenceID: ECEC6C27-612B-5D48-BEF0-6A353BC18C7B; **Location:** country: China; stateProvince: Zhejiang; locality: Xitianmushan; **Event:** verbatimEventDate: 16-18.V.1988; **Record Level:** basisOfRecord: PreservedSpecimen
- **Type status:** Other material. **Occurrence:** catalogNumber: 882682 (ZJUH); recordedBy: Chen Xuexin; individualCount: 1; sex: female; lifeStage: adult; occurrenceID: 48AFF3B7-2573-5859-97C6-0E68DCA9FB76; **Location:** country: China; stateProvince: Zhejiang; locality: Xitianmushan; **Event:** verbatimEventDate: 21.VII.1987; **Record Level:** basisOfRecord: PreservedSpecimen

#### Distribution

**Figure 19.**
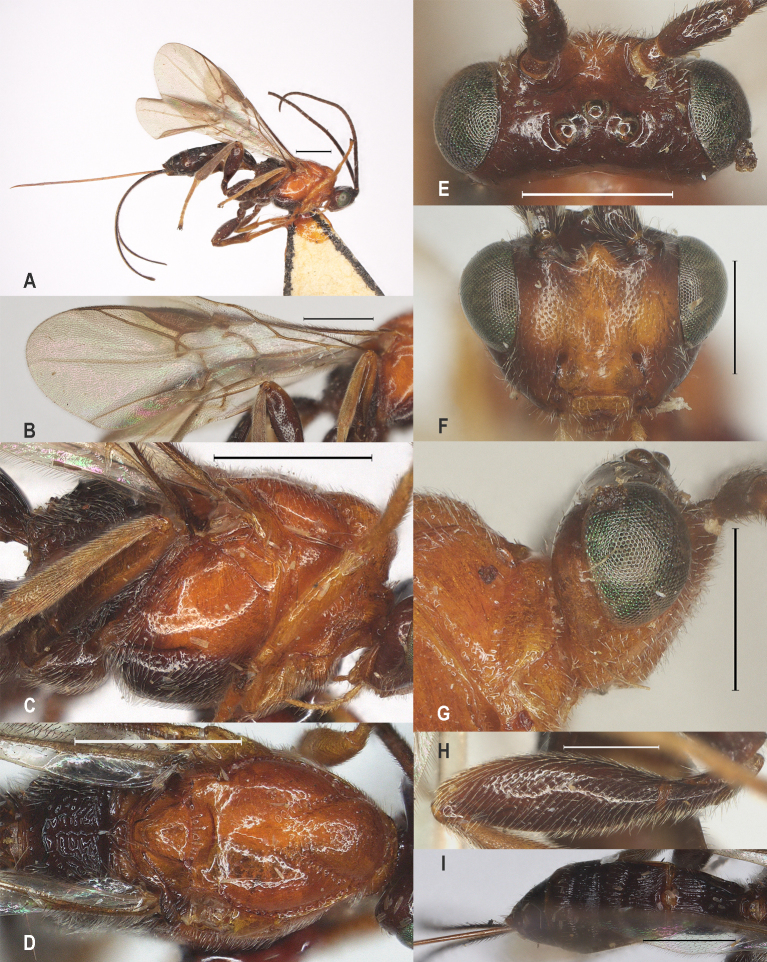
*Aerophilusromani* (Shestakov, 1940)，China. **A** habitus, lateral aspect; **B** fore wing; **C** mesosoma, lateral aspect; **D** mesosoma, dorsal aspect; **E** head, dorsal aspect; **F** head, front aspect; **G** head, lateral aspect; **H** hind femur; **I** metasoma, dorsal aspect. Scale-bars A-G, I 1 mm, H 0.5 mm.

**Figure 20.**
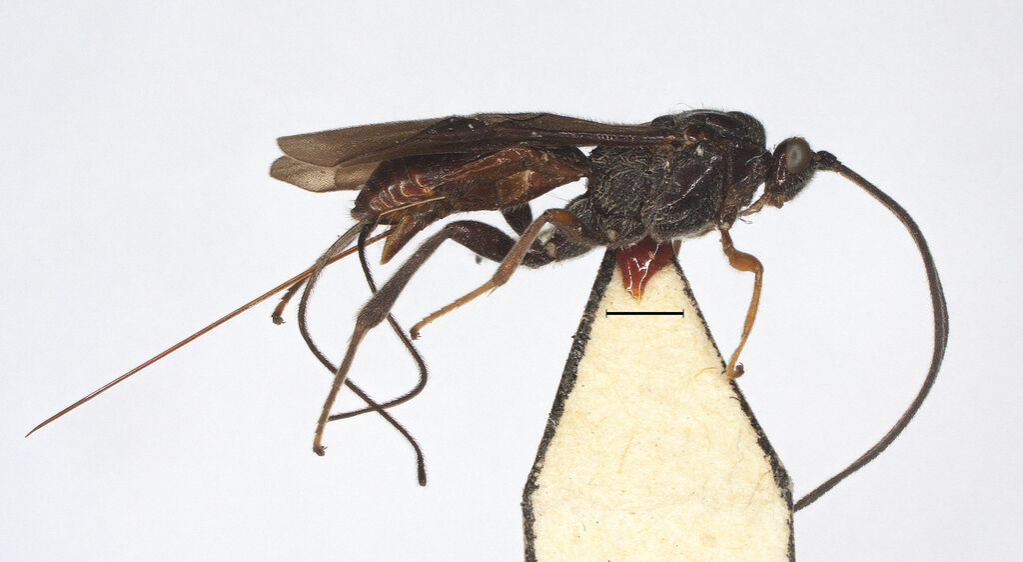
*Aerophilusromani* (Shestakov, 1940), China, variation, habitus, lateral aspect. Scale-bar 1 mm.

China (Liaoning, Guangxi, Zhejiang, Taiwan) (Figs 19, 20); Russia; Japan; Korea; India; Vietnam; Thailand.

#### Notes

*Aerophilusebulus* (Nixon, 1950) was reinstated from synonym of *Aerophilusromani* (Shestakov, 1940) by Sharkey and Clutts (2011), based on the reason that *A.ebulus* has milky-white middle and hind basitarsomeres. However, we checked the holotype of *A.ebulus* (Nixon, 1950) and found that the type actually has the middle and hind tarsi completely black, which is identical to *A.romani* (Shestakov).

### Aerophilus rufipes

(Nees, 1812)

B8F38012-724A-5764-B8A9-376C7F2223AC

Microdus rufipes Nees von Esenbeck 1812: 189.
Braunsia rufipes : *Telenga 1955*: 277; Shenefelt 1970: 375.
Agathis rufipes : Evenhuis and Vlug 1983, 1983: 122.
Bassus rufipes : Simbolotti and van Achterberg 1992: 35; Sharkey 1996: 48; Chen and Yang 2006: 57.
Braunsia germanica Enderlein 1904: 436. Synonymised by Enderlein (1908).
Bassus diversus Muesebeck 1933: 48. Synonymised by Sharkey (1996).
Microdus amurensis Shestakov 1940: 14. Synonymised by Sharkey (1998).
Lytopylus rufipes : Stevens et al. 2010: 19; Stevens et al. 2011: 6.
Aerophilus rufipes : *Sharkey et al. 2016*: 54.

#### Materials

- **Type status:** Other material. **Occurrence:** catalogNumber: 20035961 (ZJUH); recordedBy: Hu Hongying; individualCount: 1; sex: female; lifeStage: adult; occurrenceID: 1F60D8CB-AEAF-507D-A83B-98D1CDE8D7B4; **Location:** country: China; stateProvince: Xinjiang; municipality: Shihezi; locality: Dongling; **Event:** verbatimEventDate: 12.VII.2001; **Record Level:** basisOfRecord: PreservedSpecimen

#### Distribution

**Figure 21.**
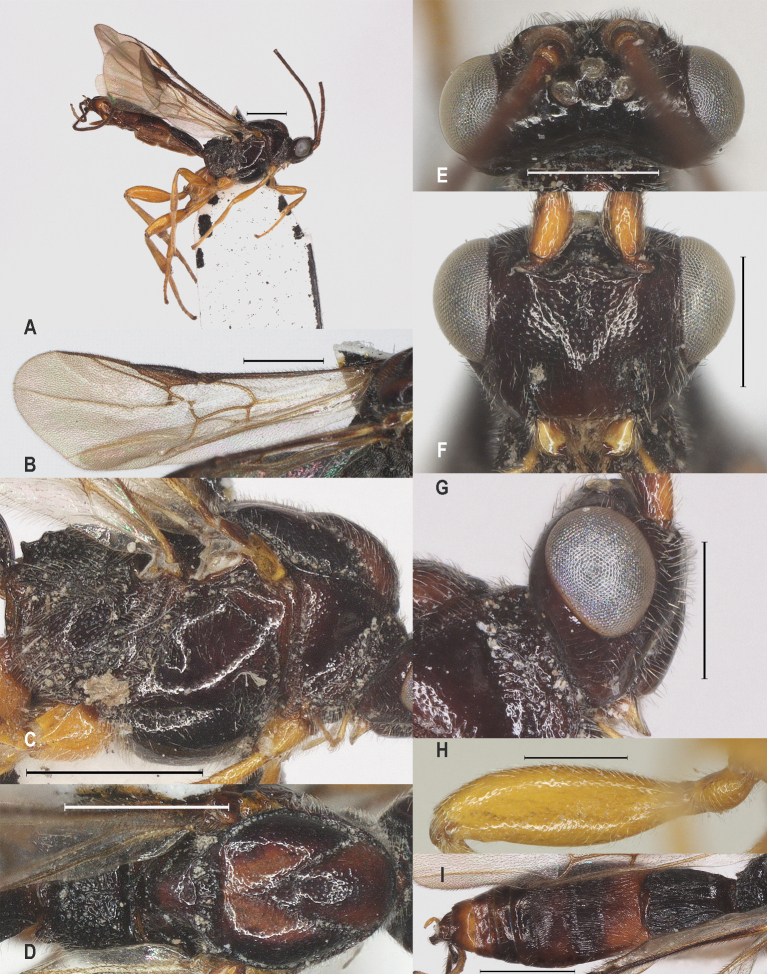
*Aerophilusrufipes* (Nees, 1812), China. **A** habitus, lateral aspect; **B** fore wing; **C** mesosoma, lateral aspect; **D** mesosoma, dorsal aspect; **E** head, dorsal aspect; **F** head, front aspect; **G** head, lateral aspect; **H** hind femur; **I** metasoma, dorsal aspect. Scale-bars A-G, I 1 mm, H 0.5 mm.

China (Xinjiang) **new record** (Fig. 21); Armenia; Austria; Azerbaijan; Belgium; Bulgaria; former Czechoslovakia; Finland; France; Georgia; Germany; Hungary; Italy; Kazakhstan; Kyrgyzstan; Lithuania; Moldova; Netherlands; Poland; Romania; Russia; Slovakia; Sweden; Switzerland; Turkmenistan; USA; Ukraine; UK; Japan; Korea.

## Identification Keys

### Key to Chinese species of the genus *Aerophilus* Foerster

**Table:** 

1	Mesoscutum not protruding forward; length of mesosoma 1.5 times its height.	2
–	Mesoscutum distinctly protruding forward (Fig. 14); length of mesosoma 1.2 times its height (Fig. 14). – China (Sichuan)	*A.convexus* Wu & Tang, sp. nov.
2	Wing membrane infuscate; vein 1-R1 of fore wing distinctly shorter than 2-R1 – China (Liaoning, Guangxi, Zhejiang, Taiwan); Russia; Japan; Korea; India; Vietnam; Thailand	*A.romani* (Shestakov)
–	Wing membrane almost hyaline; vein 1-R1 of fore wing distinctly longer than 2-R1 (Fig. 6).	3
3	Ovipositor sheath somewhat shorter than fore wing; hind leg usually yellowish-brown; length of hind femur 2.6 times their width – China (Xinjiang) new record; Armenia; Austria; Azerbaijan; Belgium; Bulgaria; (former) Czechoslovakia; Finland; France; Georgia; Germany; Hungary; Italy; Kazakhstan; Kyrgyzstan; Lithuania; Moldova; Netherlands; Poland; Romania; Russia; Slovakia; Sweden; Switzerland; Turkmenistan; USA; Ukraine; UK; Japan; Korea	*A.rufipes* (Nees)
–	Ovipositor sheath 0.5 times as long as fore wing (Fig. 9); hind leg usually mainly black (Fig. 9); length of hind femur 3.2 times its width (Fig. 7) – China (Sichuan)	*A.brevicaudis* Wu & Tang, sp. nov.

## Supplementary Material

XML Treatment for Aerophilus brevicaudis

XML Treatment for Aerophilus convexus

XML Treatment for Aerophilus romani

XML Treatment for Aerophilus rufipes
